# Making Magnetic Yeast

**DOI:** 10.1371/journal.pbio.1001274

**Published:** 2012-02-28

**Authors:** Stephanie Huang

**Affiliations:** Freelance Science Writer, Sunnyvale, California, United States of America

## Abstract

This study demonstrates that normal yeast cells can be magnetized, and identifies local redox control via carbon metabolism and iron supply as key factors involved in magnetization.

**Figure pbio-1001274-g001:**
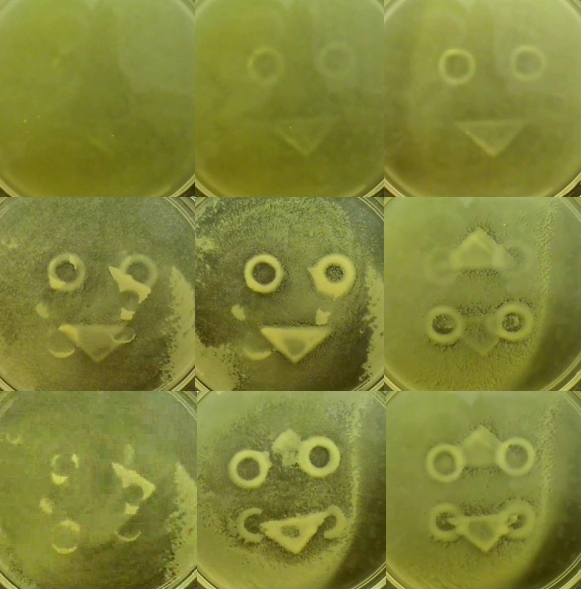
Time course of magnetic attraction of yeast (clockwise from top left). Magnetized yeast in liquid medium remain attracted to smile-shaped magnets even after rotations (at middle right and bottom left).

[Fig pbio-1001274-g001]The ability to detect and respond to magnetic fields is not a sense we always associate with living things. Yet, a number of organisms ranging from bacteria to mammals possess magnetoreception. Thanks to iron-containing structures called magnetosomes, certain species of bacteria can orient and move along the geomagnetic field. Because these bacteria generally live in water or soil, where the nutrient concentration varies with depth, navigation along geomagnetic field lines enables the bacteria to efficiently move up and down to locate nutrients. The geomagnetic field also appears to provide navigational cues to fish, turtles, birds, and other migratory animals. Magnetic particles in the nervous system may help these animals sense the magnetic field, but the biology of this mechanism is poorly understood.

Human nerve cells may also contain magnetic particles. Instead of being connected with magnetoreception, however, these abnormal iron deposits are associated with neurodegenerative diseases. Although iron is an essential element, its uptake and storage must be carefully regulated. Excess iron ions can catalyze the formation of reactive oxygen species, leading to toxic effects on the cell. Many cells regulate their internal iron concentration using a protein called ferritin, which forms a shell around iron. This shell prevents iron from causing damage to the cell and stores it for future potential use.

In a new study in *PLoS Biology*, Keiji Nishida and Pamela Silver explored the biological basis of magnetic responses by inducing magnetization in an organism that is otherwise magnet-insensitive. The researchers found that magnetization did not rely on intrinsic magnetic properties but could be induced through changes in iron storage and reduction-oxidation conditions.

Nishida and Silver chose to work with budding yeast, in which widely available genetic tools helped them modify and study the cells to understand the underlying biology of induced magnetization. Yeast do not normally produce ferritin and instead use an iron transporter to deliver excess iron to cellular storage containers called vacuoles. To boost the iron content in yeast, the researchers added iron to the cells' growth media. To determine how the storage of iron might affect magnetization, the researchers also deleted the gene for the iron transporter—which caused iron to accumulate in the cytosol—and introduced the gene for human ferritin protein—which sequestered iron in ferritin shells outside of the vacuole.

Yeast cells grown in iron-supplemented media indeed became magnetic, and the researchers showed that the cells were attracted by and moved towards a magnet. Wild-type cells (without any genetic alterations) showed the least degree of magnetization. Cells lacking the iron transporter and cells containing ferritin displayed more magnetization, and cells with both of these genetic alterations displayed the largest degree of magnetization. From these experiments, the researchers concluded that simply adding iron to growth media could induce magnetization in yeast, but that increasing the level of iron compounds stored outside the yeast vacuole enhances that magnetization.

The magnetized cells contained deposits of iron, oxygen, and phosphorous. In wild-type cells, as expected, the deposits were found in structures resembling the vacuole. In cells that lack the iron transporter, these deposits were instead found in the mitochondria, regardless of ferritin expression. The researchers speculate that conversion of iron into organic iron compounds within the mitochondria may help explain the larger degree of magnetization of cells that lack the iron transporter compared to the wild-type.

Nishida and Silver conducted further experiments to determine which signaling pathways contributed to the induced magnetization of their yeast strains. Through a series of genetic experiments, they identified the protein Tco89p, a component of the TORC1 protein complex. TORC1 is an important signaling complex that integrates stimuli from nutrient, cellular stress, and reduction-oxidation conditions in order to control cell growth. Reduction-oxidation refers to chemical reactions in which atoms transfer electrons between one another. The researchers found that Tco89p promoted an oxidative state in the cell, which induced the formation of iron-containing particles and subsequent magnetization of the cells. Extending their findings, they also identified other genes involved in carbon processing and reduction-oxidation regulation that contributed to induced magnetization.

This study shows how magnetization might be induced in organisms. Even cells without intrinsic magnetic properties can become magnetized through changes to existing pathways of iron storage and regulation of reduction-oxidation conditions.

The study provides a compelling example of the power of transferring molecular systems from difficult-to-study organisms, like humans, into organisms that can be easily manipulated to examine the molecular underpinnings of a given process. Inducing magnetization in yeast, for example, enabled these researchers to tease apart signaling pathways that contribute to magnetization, reduction-oxidation regulation, and iron storage. These results and further studies could lead not only to a better understanding of how magnetic particles and structures enable detection of magnetic fields, but also how magnetic particles function in neurodegenerative diseases. Induced magnetization also represents an exciting avenue for bioengineers who could engineer cells that respond to magnetic fields or modulate their own magnetization. This would enable selective measurement or manipulation of these cells using magnetic fields, which may be helpful in tracking diseased tissue or delivering therapeutic cells to disease sites.

**Nishida K, Silver PA (2012) Induction of Biogenic Magnetization and Redox Control by a Component of the Target of Rapamycin Complex 1 Signaling Pathway. doi:10.1371/journal.pbio.1001269**

